# Melanoma Immunotherapy: Next-Generation Biomarkers

**DOI:** 10.3389/fonc.2018.00178

**Published:** 2018-05-29

**Authors:** Sabrina A. Hogan, Mitchell P. Levesque, Phil F. Cheng

**Affiliations:** ^1^Department of Dermatology, UniversitätsSpital Zürich, Gloriastrasse, Zurich, Switzerland; ^2^Faculty of Medicine, Universität Zürich, Zürich, Switzerland

**Keywords:** melanoma, immunotherapy, biomarkers, next-generation sequencing, review literature as topic

## Abstract

The recent emergence of cancer immunotherapies initiated a significant shift in the clinical management of metastatic melanoma. Prior to 2011, melanoma patients only had palliative treatment solutions which offered little to no survival benefit. In 2018, with immunotherapy, melanoma patients can now contemplate durable or even complete remission. Treatment with novel immune checkpoint inhibitors, anti-cytotoxic T-lymphocyte protein 4 and anti-programmed cell death protein 1, clearly result in superior median and long-term survivals compared to standard chemotherapy; however, more than half of the patients do not respond to immune checkpoint blockade. Currently, clinicians do not have any effective way to stratify melanoma patients for immunotherapies. Research is now focusing on identifying biomarkers which could predict a patient’s response prior treatment initiation (or very early during treatment course), in order to maximize therapeutic efficacy, avoid unnecessary costs, and undesirable heavy side effects for the patient. Given the rapid developments in this field and the translational potential for some of the biomarkers, we will summarize the current state of biomarker research for immunotherapy in melanoma, with an emphasis on omics technologies such as next-generation sequencing and mass cytometry (CyTOF).

Immunotherapy has revolutionized the management of metastatic melanoma. Prior to 2011, the median survival for metastatic melanoma was 9 months, compared to greater than 18 months in 2017 (1). Patients now benefit from novel immune checkpoint inhibitors (ICIs), anti-cytotoxic T-lymphocyte protein 4 (CTLA-4) and anti-programmed cell death protein 1 (PD-1). From the latest survival data of the Checkmate 067 trial, progression-free survival (PFS) for ipilimumab is 2.9 months, for nivolumab 6.9 months, and for the combination of nivolumab and ipilimumab 11.5 months. Overall survival (OS) of the ipilimumab group was 19.9 and 37.6 months for the nivolumab group. Median OS was not reached in the combination nivolumab and ipilimumab group with a minimum follow-up time of 36 months (2–6). Although OS is extended, not all patients benefit from immunotherapy. Response rates for ipilimumab range from 11% to 19% (4, 5) and for pembrolizumab or nivolumab from 33% to 44% (2, 6, 7). These new ICIs clearly show superior median and long-term survivals compared to standard chemotherapy; however, more than half of the patients do not respond to immune checkpoint blockade. Currently, there are no clinically approved biomarkers to aid in patient selection in melanoma. In this review, we seek to delineate the current state of biomarker research for immunotherapy in melanoma, with an emphasis on omics technologies such as next-generation sequencing (NGS) and mass cytometry (CyTOF). Given the urgent clinical need for such biomarkers, we decided to focus on human studies only, which we think are more clinically relevant.

## Immune Checkpoints

**Figure 1:**
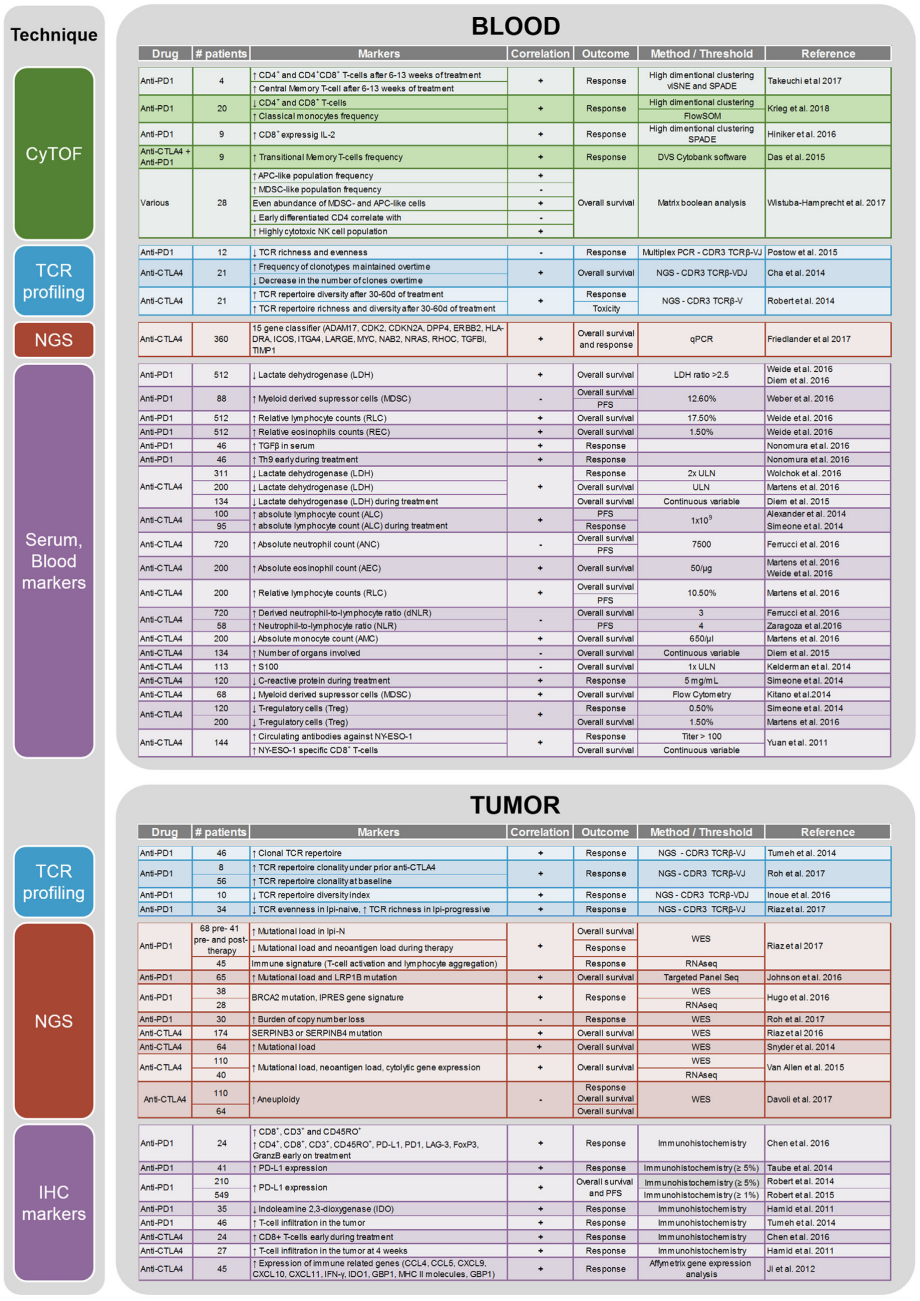
Summary of the biomarker studies performed on peripheral blood and tumor on anti-CTLA4 and anti-PD1 treatment.

CTLA-4 and PD-1 are two immune checkpoints regulating immune homeostasis. CTLA-4 is a negative regulator of T-cell priming that acts to control naïve T-cell activation by competing with the co-stimulatory molecule CD28 for binding to shared ligands CD80 and CD86 on antigen-presenting cells (APCs) in the lymph node (8). Ipilimumab, a monoclonal antibody against CTLA-4, was the first agent approved for the treatment of unresectable or metastatic melanoma that showed an OS benefit in a randomized phase III trial (4). PD-1 is a T-cell exhaustion marker which is upregulated by T-cells upon activation during priming or expansion and binds to one of two ligands: programmed cell death 1-ligand 1 (PD-L1) and -ligand 2 (PD-L2) (9–11). Pembrolizumab and nivolumab are monoclonal antibodies against PD-1 that have both shown OS benefit in randomized phase III trials and are approved for the treatment of metastatic melanoma (2, 7). Furthermore, nivolumab and pembrolizumab have both improved OS compared with ipilimumab in metastatic melanoma patients that are naïve to both agents. Combination therapy with ipilimumab and nivolumab has demonstrated additional clinical activity with objective response rates ranging from 50% to 60% and improved OS compared to ipilimumab alone. Although ipilimumab, nivolumab, and pembrolizumab have significantly improved the survival of melanoma patients, there are major toxicities associated with the use of these drugs [reviewed in Ref. (12)]. Grade 3 and higher adverse events are seen in about 20% of patients treated with ipilimumab, in 15% of patients treated with nivolumab, and in 50% of patients treated with the combination of both drugs (6). As these therapies result in objective responses for only a subset of patients, there is a crucial need to identify biomarkers that can potentially predict the efficacy of anti-CTLA-4 or anti-PD-1 treatment or identify a specific subset of patients who may benefit from immunotherapy. A summary of current potential biomarkers for immunotherapies in metastatic melanoma patients is listed in Figure 1.

## Clinical Biomarkers

Approved markers for melanoma monitoring have not substantially evolved over the past decade. Clinicians have mainly used the TNM staging system as a diagnostic and prognostic indicator. In 2009, lactate dehydrogenase (LDH) was shown to be an independent predictor of survival in melanoma and was therefore added to the AJCC guidelines (13). Accelerated metabolism in cancer cells requires increased glycolysis that creates a high amount of LDH as a byproduct, which is therefore a robust proxy to assess tumor burden (14). It is the only accepted serum biomarker with prognostic value for OS in melanoma (15). In the context of immunotherapies, elevated LDH is a negative prognostic marker for patients treated with ipilimumab (16) and with pembrolizumab (17, 18). However, subgroup analysis of anti-PD-1 treated cohorts recently pointed out that LDH level is not correlated with the duration of response (KEYNOTE-006). Indeed, once patients show response to the treatment, the LDH level is not associated with the duration of the remission period. As described by Diem et al. in a study specifically assessing the role of LDH as a marker for anti-PD-1 therapy, LDH is nevertheless a useful marker to monitor disease progression and help treatment decisions (19).

Another well-known marker to monitor melanoma is S100, which is a good indicator of advanced clinical disease stage (20). S100 was shown to be predictive of response to anti-CTLA-4 (16). However, similar to LDH, S100 seems to mainly be a proxy of disease stage, able to highlight very ill patients who are more unlikely to respond to the treatment due to the high tumor burden of the disease, but not actually able to predict response to immunotherapies. The same is true for the number of organs involved, which was another potential marker, proposed by Diem et al., to stratify patients prior to anti-CTLA-4 therapy (21).

C-reactive protein (CRP) was described as a negative prognostic factor for anti-CTLA-4 treatment (22). Unlike LDH and S100, CRP is directly related to immune response. However, it is a general marker of inflammation and is not specific to melanoma, ergo, an increase in CRP levels may also be the result of any other ongoing infection (23). For anti-PD-1 therapy, intra-tumoral PD-L1 expression, evaluated by immunohistochemistry, has been assessed as a predictive biomarker. The results have been inconclusive due to a lack of standards for PD-L1 “positivity”. Different antibodies and different evaluation criteria have been used for PD-L1 expression in clinical trials. Some studies have used a >5% cutoff (Checkmate-066 and Checkmate-067), whereas others have used >1% cutoff (KEYNOTE-006). In the Checkmate-066 trial, both PD-L1 negative and positive patients had better outcomes than chemotherapy-treated patients, suggesting that PD-L1 status was not a relevant stratification marker (2). More research will be needed to standardize the assessment of PD-L1 expression for it to become a biomarker for anti-PD-1 therapy in melanoma. Blood markers which hold the most potential toward predicting response to immunotherapies are immune cell populations. Indeed, they are either themselves part of or directly influencing the immune response against the tumor. The different findings related to blood cytology as a biomarker for immunotherapy in melanoma are summarized in Figure 1. Briefly, for anti-CTLA-4 treatment, absolute neutrophil count, absolute lymphocyte count, neutrophil to lymphocyte ratio, absolute eosinophil count, relative lymphocyte count (RLC), absolute monocyte count, antibodies against NY-ESO1, T-regulatory cell count, and myeloid-derived suppressor cell (MDSC) count have been described as predictive biomarkers. In anti-PD-1-treated patients, RLC, relative eosinophil count (REC), and MDSC count seem to hold some predictive potential prior to treatment initiation. In addition, increased serum levels of TGFβ and increased frequency of Th9 cells in the peripheral blood were detected in responders to nivolumab prior to therapy initiation (24). Unfortunately, most of the studies were performed on small cohorts and the results have not been verified in larger prospective trials (25). Although guidelines have been published about how to best perform biomarker studies (26), most research groups have different evaluation criteria. In this review, we sought to document the most relevant biomarkers associated with immunotherapy outcome in melanoma patients. For a systematic review of clinical biomarkers, see Ref. (18).

## Biomarkers from Next-Generation Sequencing

Whole exome (WES) and RNA sequencing (RNAseq) are powerful tools for evaluating the genomic landscape of a tumor. WES only captures the exonic gene regions, so it enriches for mutations in coding regions, while RNAseq can provide the entire transcriptome of a sample and is useful for establishing gene signatures for specific cohorts within a patient group. In terms of immunotherapy, WES has been useful in determining mutational load and discovering neoantigens in melanoma tumors (27). Melanoma has one of the highest mutation rates of all cancers (28–30) and has a high probability for neoantigen generation. Neoantigens are the result of somatic mutations which translate into a mutated protein that is detected and presented by APCs. Neoantigens are an attractive target for immunotherapy as they are only expressed by the tumor and not by the normal tissue. Many studies have utilized WES and RNAseq to evaluate the mutation profile and gene expression changes in patients treated with anti-CTLA-4 and anti-PD-1, with the aim to find biomarkers to predict response.

Snyder et al. performed WES on 64 patients treated with anti-CTLA-4 (31). This study was the first to associate mutational burden to clinical benefit and they also defined a neoantigen signature associated with clinical benefit. They concluded that, although patients with high mutation burden are more likely to respond to anti-CTLA-4, the types of neoantigen the patient expresses will ultimately determine their response. Van Allen et al. performed WES on 110 patients and RNAseq on 40 patients treated with anti-CTLA-4 (32). They could confirm that mutational load is associated with clinical benefit to anti-CTLA-4 treatment. Neoantigen load was also measured and this parameter was also significantly associated with response; however, they could not detect the neoantigen signature seen in the study performed by Snyder and colleagues. They concluded that clinically beneficial neoantigens are most likely private events (specific to each individual) and recurrent neoantigens (consistent in the general population) are quite rare. Van Allen and colleagues also analyzed the transcriptome in a subset of these patients and found that expression of cytolytic markers, such as granzyme A and perforin, were beneficial for response. Expression of CTLA-4 and PD-L2 was also associated with clinical benefit. Riaz et al. performed WES on 174 patients treated with anti-CTLA-4 therapy (33). They discovered 48 patients with mutations in SERPINB3 or SERPIN4 and observed that those patients were more likely to be responders. Patients with SERPINB3 or SERPINB4 mutations also had higher mutational loads. Friedlander et al. performed a quantitative polymerase chain reaction (PCR) study on peripheral blood from 360 patients receiving anti-CTLA-4 therapy (34). From a panel of 169 genes, they established a 15 gene signature that was predictive and prognostic for response and 1-year OS to anti-CTLA-4 treatment. The 15 genes are ITGA4, LARGE, CDK2, TIMP1, DPP4, NRAS, ERBB2, NAB2, ADAM17, RHOC, TGFB1, CDKN2A, HLADRA, MYC, and ICOS.

In order to elucidate resistance mechanisms and biomarkers of response to treatment, Hugo et al. used WES (38 patients) and RNAseq (28 patients) on a set of melanoma patients treated with anti-PD-1 (35). Mutational load did not have a significant association to response to anti-PD-1 therapy and neoantigen load was not significantly correlated with response either. Nonetheless, mutational load was associated with OS suggesting that other factors influence response to anti-PD-1 and survival. BRCA2 mutations occurred in 30% of the responders to anti-PD-1. RNAseq analysis uncovered a co-enrichment of 26 gene signatures in 9 of the 13 non-responding patients, which the authors termed innate anti-PD-1 resistance (IPRES) signature. They validated the IPRES signature on three other datasets and found over-representation in anti-PD-1 non-responding samples.

In a small study of four patients treated with anti-PD-1, Zaretsky et al. used WES on patients that developed new lesions under anti-PD-1 therapy. They discovered that the progressive tumors acquired JAK1, JAK2, or B2M loss of function mutations. JAK1 and JAK2 mutations cause insensitivity to interferon gamma-induced arrest and B2M mutations led to a loss of MHC class 1 expression (36). In a follow-up study, Shin et al. performed WES on 23 patients before anti-PD-1 treatment (37). In their cohort, mutational load had no association to response and one of the non-responders had a loss of function mutation in JAK1. This study confirms the role of JAK1 as a marker for innate and adaptive resistance to anti-PD-1, although it might be a rare occurrence.

Roh et al. performed WES on sequential biopsies of patients treated with anti-CTLA-4 and then anti-PD-1. Thirty patients had WES at baseline, 3 on anti-CTLA-4 treatment, 25 at anti-CTLA-4 progression, 18 on anti-PD-1 treatment, and 12 at anti-PD-1 progression. Overall, they found that mutation burden was not associated with response, but high copy number loss was associated with poor response (38). In the regions with recurrent copy number loss, PTEN was one of the notable tumor suppressor genes suggesting that it could be a driver of resistance mechanisms to immunotherapy. Another study also observed that PTEN loss was associated with resistance to anti-CTLA-4 therapy (39).

Johnson et al. performed targeted panel sequencing on 65 patients treated with anti-PD-1 therapy. In their cohort, mutational load was associated with response to anti-PD-1 and patients with high mutational load (>23.1 mutations/MB) had longer PFS and OS compared to the intermediate mutational load group (3.3–23.1 mutations/MB) and the low mutation load (<3.3 mutations/MB) group. They observed more frequent BRCA2 mutations in responders than in non-responders (5/32 vs. 2/33). LRP1B mutations were significantly enriched in the responder group (11/32) compared to the non-responder group (1/33). LRP1B mutated patients also had a higher mutational load compared to LRP1B wild-type patients.

Riaz et al. performed WES on 68 patients treated with anti-PD-1 that had previously progressed on anti-CTLA-4 therapy (35 patients) or were naïve to anti-CTLA-4 (33 patients). Mutational load was associated with clinical benefit in the anti-CTLA-4-naïve group, but not the anti-CTLA-4-resistant group. No single gene mutations were significantly associated with response or resistance to therapy. Decreased mutational and neoantigen load during therapy was associated with response in both anti-CTLA-4-naïve and anti-CTLA-4-resistant groups. RNAseq analysis of the pretreatment samples showed an enrichment in T-cell activation and lymphocyte aggregation pathways. These signatures indicate an immunologically active tumor, or “hot tumor,” and all patients with complete response or partial response in the anti-CTLA-4-resistant group had the “hot tumor” signature, although not all responders in the anti-CTLA-4-naïve group had the “hot tumor” signature. Riaz et al. also investigated the early effects of anti-PD-1 treatment (29 days after start) by RNAseq and uncovered a global increase in immune checkpoint genes such as PD-1, CTLA-4, CD274 (PD-L1), ICOS, and LAG3 in all samples. For responders, the significant pathways included inflammatory response and cytokine-mediate signaling pathways.

Finally, Davoli et al. investigated the role of aneuploidy in response to immunotherapy (40). They analyzed the copy number data from 5,255 tumor/normal samples, representing 12 cancer types from The Cancer Genome Atlas project, and found that for most tumors, there was a positive correlation between aneuploidy severity and mutational load. They also found that tumors with high levels of aneuploidy showed elevated expression of cell cycle and cell proliferation markers, as well as a reduced expression of markers for cytotoxic immune cell infiltrates. Aneuploidy levels were a stronger predictor of markers of cytotoxic immune cell infiltration than tumor mutational load. To correlate aneuploidy with response to immunotherapy, they used data from Snyder et al. and Van Allen et al. (31, 32) and found in both datasets that high levels of aneuploidy correlated with poorer survival.

## Biomarkers from T-Cell Receptor (TCR) Profiling

Antigen detection by T-cells is by definition dependent on tumor-specific T-cell generation and clonal amplification. In the context of immunotherapies, which aim at enhancing the recognition of the cancer cell by the immune system, there is an obvious rational basis for examining the T-cell repertoire in order to shed light on the specific mode of action of the drug and find potential biomarkers of response.

The main challenge when analyzing TCR repertoire is its immense diversity. The TCR is a heterodimer comprised of two chains αβ or δγ. The β and δ chains, are generated by the random rearrangement of a variable region (V), a diversity region (D), and a joining region (J) with a constant region (C). The α and γ chains, consists of segments from the V, J, and C regions. Additional complexity is introduced by random addition or deletion of nucleotides at the junction sites of V, D, and J. The theoretical limit of the TCR repertoire is in the range of 10^15^, which is several magnitudes higher than the total amount of T-cells in the body, approximately 4 × 10^11^ (41). The estimated number for the TCR repertoire is in the order of 10^6^ to 10^8^ (42). Most of the studies mainly assess the complementarity-determining region 3 (CDR3) from β chain, which is considered an acceptable proxy for estimating diversity since it is the most variable region of the receptor and that αβT-cells represent about 90% of all T-cells (43). Due to the advances in NGS, it is possible now to identify each individual TCR sequence in the CDR3 region (44). Multiplex PCR is one of the widely used methods to amplify the CDR3 region. Primers for the J alleles or the constant region of the TCR α and β chains are used together with a mix of primers for all known V alleles. A drawback of multiplex PCR is that it is limited to known V alleles. As a result, for TCR discovery experiments, other methods such as targeted enrichment—a technique where RNA baits capture the TCR receptor, usually the CDR3 region—are preferred. Since bait capture takes into account mismatches, it allows for discovery of new alleles and TCR receptors. In the context of immunotherapy, TCR repertoire analysis is useful for determining if the tumor-reactive clones have undergone activation and clonal expansion. In an adequate immune response, the tumor-specific T-cells will represent a significant proportion of the whole repertoire and therefore be assessable at the level of the whole TCR population.

In 2014, Robert et al. compared pre- to post-treatment peripheral blood mononuclear cell (PBMC) samples from melanoma patients treated with anti-CTLA-4 (45). The results from deep sequencing of the multiplex PCR for the TCR Vβ CDR3 region showed that 19 out of 21 patients had an increased number of unique clonotype (richness). There was no significant difference in the V or J segment usage and no difference in the total of unique sequences between responders and non-responders. The number of unique productive sequences in the top 25% of clones showed a particularly high increase in diversity after treatment. Those changes were not associated with peripheral lymphocyte count; however, CD8^+^ tumor-infiltrating cells showed a positive correlation with the TCR repertoire diversity. Finally, they showed that patients experiencing more toxicities had more diverse sequences post treatment. Overall, this study reports that anti-CTLA-4 treatment increases TCR repertoire diversity in an unspecific manner. Subsequently, the same group performed a similar study on 9 anti-PD-1-treated patients and compared the results (46). Unlike the effect of anti-CTLA-4, anti-PD-1 therapy does not increase TCR repertoire complexity, on the contrary, 4/9 samples show a decrease >15% in the absolute number of unique sequences and only one had an increase >15%. Those results suggest that the mode of action of the two drugs is considerably different.

Cha et al. shed more light on the potential mechanism of anti-CTLA-4; their study assessed the changes in TCR repertoire between baseline and 4 weeks of treatment in PBMCs from 21 melanoma patients (47). They confirmed that anti-CTLA-4 treatment induces a significant change in the clonotypes frequency compared to healthy donors. They showed that the diversification is the result of a higher gain of new clonotypes and lower loss of existing ones. The number of therapy-induced expanding clones are not different between the responders and the non-responders, which is in line with what Robert et al. described. However, patients who survived longer exhibited less clonotypic changes overtime, they maintained the most abundant clones which were present at baseline and also had fewer clones significantly decreasing in frequency. Finally, they also demonstrated that the clones expanding in response to therapy are largely non-naïve T-cells, suggesting that patients who respond to the therapy already have pre-primed T-cells in circulation before the onset of anti-CTLA-4 treatment.

In light of these findings, Postow et al. hypothesized that the shape of the TCR repertoire prior to treatment initiation may influence the likelihood of a response to the treatment (48). Twelve baseline PBMC samples from 4 responders and 8 non-responders to anti-CTLA-4 were analyzed for richness and evenness of the TCR repertoire. In this small cohort, they first showed that patients who responded to the treatment have more similar VJ usage among each other than compared to the VJ usages in the non-responders. Furthermore, low richness or evenness of the TCR repertoire was significantly associated with a poor response to anti-CTLA-4. That is, a TCR repertoire composed of less unique clones (less diverse) or skewed toward a few specific clones (very clonal) is predictive of a non-response to the treatment.

The detection of clonally expanded tumor-specific T-cell in the blood indicates that the immune system mounted an immune response against a foreign entity, which could be the tumor. However, it is not certain that the activated T-cells would be able to home to the tumor efficiently and be able to kill the cancer cells. Therefore, it is also important to assess the immune status at the tumor site.

Tumeh et al. performed a very elegant study exploiting tumor samples from melanoma patients prior and during anti-PD-1 treatment (49). By qualitative and quantitative immunohistochemistry, they revealed that, at baseline, patients who eventually respond to the therapy, have more CD8^+^ T-cells at the invasive margin of the tumor compared to non-responders. This population increases and migrates toward the center of the tumor during treatment in responders. They showed that an efficient response to anti-PD-1 therapy requires pre-existing CD8^+^ T-cells, which are most likely tumor specific. To confirm this theory, they sequenced the TCR Vβ region of tumor baseline samples and found that responders indeed had a more clonal TCR repertoire. On treatment, samples of responders showed significantly more clonal expansion than non-responders.

Johnson et al. used NGS to assess differences between baseline samples of responders and non-responders to anti-PD-1 therapy (50). They assessed mutational load as well as specific mutations differentially occurring in responders and non-responders. They also investigated the TCR repertoire clonality of 42 samples and did not find any association with response. However, it is important to mention that times of sample acquisition were not immediately before and after treatment. The timing was quite broad, the study allowed the inclusion of samples collected over 12 months before start of treatment and also after treatment initiation. When the analysis was performed only on samples obtained within 4 months of treatment initiation, the non-responder group was only represented by five samples, including one potential outlier. Nonetheless, they noticed a trend toward higher clonality at baseline in patients who eventually responded to the therapy.

Inoue et al. analyzed the TCR repertoire of 10 pre- and post-anti-PD-1 tumor samples (51). They noticed that the clonotypes with a read frequency >0.5% at baseline significantly increased after treatment in responders. The calculation of the diversity index highlighted a slight decrease in tumors of responders compared to non-responders, which suggests oligoclonal expansion of certain TCR clones.

More recently, Roh et al. published a complementary analysis on a cohort of patients for which they performed TCR sequencing (38). They analyzed tumor samples from melanoma patients treated sequentially with anti-CTLA-4 and anti-PD-1 *via* WES and TCR sequencing. The TCR clonality assay revealed that there was no significant difference between responders and non-responders, pre- or on-treatment with anti-CTLA-4. A subpopulation of the patients (*n* = 8) received anti-CTLA-4 followed by anti-PD-1 after progression. All three responders to anti-CTLA-4 followed by anti-PD-1 showed an increase in TCR clonality during anti-CTLA-4 treatment. In addition, higher TCR clonality was seen in the responders prior to treatment and on treatment with anti-PD-1.

Riaz et al. investigated the evolution of melanoma tumors and their microenvironment under anti-PD-1 therapy (52). Patients who had previously progressed on anti-CTLA-4 and were naïve to anti-CTLA-4 were included in the study. They performed TCR sequencing on 34 samples pre- and 4 weeks post- anti-PD-1 treatment. There were no statistically significant differences in the baseline samples of either group. On anti-PD-1 therapy, the anti-CTLA-4-pretreated group had increased TCR richness associated with response, whereas the anti-CTLA-4-naïve group had decreased TCR evenness associated with response. In line with Roh et al., pretreatment with anti-CTLA-4 seems to increase the expansion of tumor-specific T-cell cells, which are additionally expanded during anti-PD-1 treatment.

## Mass Cytometry (CyTOF)

The advent of CyTOF has allowed a more comprehensible analysis of the whole immune system and will be an important asset for immune oncology (53). The basic principle of CyTOF is similar to conventional flow cytometry. The assay quantifies multiple protein expression markers at the single-cell level. In contrast to flow cytometry, the detection is not achieved by fluorophore excitation, but by stable mass isotope quantification. The transition isotope bound to the antibodies are analyzed by a time of flight mass spectrometer. CyTOF has some advantages over flow cytometry, namely, the high purity of the metal isotopes reduces background noise, eliminating spectral spillover and cellular autofluorescence associated with conventional flow cytometry. It also enables the detection of more markers in the same experiment, theoretically up to a hundred. Multiple samples can be analyzed at the same time thanks to a barcoding strategy (up to 20), and therefore reduce inter-sample variation. CyTOF has primarily been used to analyze peripheral blood from patients undergoing immunotherapy. A better characterization of the precise mode of action of those drugs is crucial to help overcoming and predicting resistance as well as contributing to optimal development of future combination therapies.

In 2015, Das et al. analyzed peripheral blood from melanoma patients undergoing immunotherapy with anti-CTLA-4, anti-PD-1, or the combination of the two (54). Samples were collected at baseline and after 3 weeks of treatment. In this early study, CyTOF was mainly used to further characterize the cell population of interest previously identified by flow cytometry. The analysis revealed that the Ki67^+^ cells, increasing after combination treatment, have a transitional memory T-cell-like phenotype. Additional experiments were performed using other techniques than CyTOF, which lead the authors to conclude that anti-PD-1 and anti-CTLA-4 have distinct effects on the immune system.

In the context of a clinical trial assessing the safety of combining radiotherapy and immunotherapy in melanoma patients, Hiniker et al. analyzed baseline and follow-up PBMC samples from 9 patients (3 progressive disease, 6 complete response/partial response) (55). CyTOF analysis revealed that the level of CD8^+^ T-cells expressing IL-2 were higher at baseline and in the follow-up samples of responding patients. The same was true for central memory CD8^+^ T-cell levels. However, the cytokine production was not significantly different from the population seen in non-responding patients, thereby suggesting that the cells are not functionally different from the non-responders.

The first study to use CyTOF as a main technique for analyzing human melanoma patient samples was performed by Wistuba-Hamprecht et al. in early 2017 (56). The analysis consisted in performing CyTOF on 28 PBMC samples from stage IV melanoma patients who received different courses of treatments. A higher frequency in the APC-like population had a positive association with OS, whereas a higher frequency in the MDSC-like population showed negative association with OS. Overall, an equal abundance of MDSC- and APC-like cells is associated with better survival. The analysis of the T-cell compartment revealed that there was a clear interpatient heterogeneity in the CD4^+^ and CD8^+^ T-cell compartments compared to the other compartments which have more homogenous frequencies between patients. Only one αβT-cell population had some prognostic potential: a higher level of early differentiated CD4^+^ T-cell was correlated with poorer OS. In the natural killer cell compartment, a highly cytotoxic cell population tends to correlate with better OS. Finally, a comprehensive analysis of immune signatures of all the melanoma-associated phenotypes identified a specific cluster with high prognostic capacity, performing even better than LDH. This cluster is significantly associated with poor OS and represented by an overall lower diversity across all the compartments, and especially in the myeloid compartment.

Takeuchi et al. investigated the effect of immunotherapy in melanoma patients by comparing PBMCs from 4 different patients receiving anti-PD-1 (2 responders and 2 non-responders) (57). The panel was composed of 35 markers and they used high-dimensional clustering to analyze the data. The main finding in this paper is that CD4^+^ and CD4^+^CD8^+^ cell populations increase during therapy. CD4^+^CD27^+^FAS^−^ central memory T-cell were shown to expand in a higher proportion in responders than in non-responders. These results were validated in a separate cohort (*n* = 4).

More recently, Krieg et al. performed a comprehensive analysis, assessing the correlation between baseline peripheral immune signature and response to anti-PD-1 in melanoma patients (58). The cohort was composed of 20 patients from whom baseline and on treatment samples were obtained. They used an optimized immune marker panel and a customized, interactive bioinformatics pipeline in order to identify potential predictive biomarkers. Three different CyTOF panels were used: one for the phenotypic characterization of lymphocytes, one to assess the T-cell functions, and the third one to characterize monocytes, which consisted of 30, 26, and 25 markers, respectively. By performing hierarchical clustering of all the samples pooled together, they identified a differential marker expression in responders compared to non-responders. Further analysis, and validation in an independent, blinded cohort by conventional flow cytometry, revealed that, at baseline, responders had a higher frequency of classical monocytes and lower frequency of lymphocytes compared to the non-responders.

## Outlook

The development of high-throughput technologies such as NGS and CyTOF have allowed researchers and clinicians to evaluate hundreds to tens of thousands of genes from a bulk tumor to a single-cell level. NGS is an invaluable tool for analyzing mutations and copy number profiles, gene expression changes and gene signatures, epigenetic alterations, the TCR repertoire, and single-cell gene expression changes. The recent development of CyTOF has also allowed the analysis of many markers at a single-cell level. In the context of immunotherapy, these high dimensional datasets will enhance the discovery of novel biomarkers, prognostic markers, and resistance mechanisms.

Next-generation sequencing biomarker discovery for anti-CTLA-4 treatment have uncovered that mutational load and neoantigen load are the most informative for response and OS, but they are not perfect biomarkers as some non-responders may also present with high mutational load. Aneuploidy could also help foresee response to anti-CTLA4 since it was highlighted as an independent predictor in a multivariate Cox model which included mutational load. Copy number analysis could as well be informative as loss in chromosome 10 was shown to be a poor prognostic marker in two studies. Many of these studies also analyzed tumor samples upon progression and found no recurrent genetic mutation, which could mean that resistance to anti-CTLA-4 is patient specific. In the context of anti-PD-1 treatment, mutational load is not a clear informative marker for response. As anti-PD-1 is a relatively new therapy, no large cohort studies with over 100 patients for NGS biomarker discovery have yet been performed. There are single patient examples showing that genes involved in the JAK–STAT pathway or antigen presentation could be predictive biomarkers for anti-PD-1 treatment. Loss of function mutations in JAK1, JAK2, and B2M are negative biomarkers for response and are involved in resistance to anti-PD-1 treatment in individual cases, but these mutations do not seem to be recurrent. RNAseq analysis from several studies suggest that tumors with high immune activity are more likely to respond to anti-PD-1. To better stratify patients for anti-CTLA-4 or anti-PD-1 treatment, a combinatorial approach investigating WES, copy number variation, and RNAseq would be needed.

Overall, most studies support that anti-CTLA-4 and anti-PD-1 modulate TCR repertoire clonality upon treatment. This strengthens the notion that tumor-specific T-cell populations are affected by CTLA-4 or PD-1 inhibition. In summary, most studies support that anti-CTLA-4 induces an expansion of clones in a non-specific manner and, therefore, broadens the TCR repertoire. On the other hand, anti-PD-1 seems to favor the proliferation of fewer specific clones giving rise to a more skewed repertoire, thereby suggesting that the baseline TCR repertoire of the patients plays a role in the response to the treatment. However, for the moment, those predictions arise mainly from early on-treatment evaluations that examined the evolution of the repertoire from baseline, as we are not yet able to precisely pinpoint the tumor-specific clones that, once clonally expanded, will facilitate tumor elimination. It is also important to highlight that the mode of action of the current immunotherapies are still debated and we do not fully comprehend their overall impact on different immune cell subpopulations. As a result, it is difficult to assess the global impact of the drugs on the immune response by investigating specific mechanisms individually. This is why high-throughput techniques discussed here are powerful emerging tools, which will allow us to elucidate this problem by looking at numerous markers simultaneously. The more we increase our knowledge of exact mechanisms, the better we will be able to exploit the therapies by using them in a targeted/patient-specific manner. Interesting work by Twyman-Saint et al. combining anti-CTLA-4, anti-PD-1 and radiotherapy, underpins this assertion (59).

To our knowledge, despite the great potential held by CyTOF technology, to date, no research was published on the analysis of human melanoma tumor samples in the context of immunotherapies. One should however expect to see more forthcoming data, thanks to a novel exciting add-on technology that is starting to emerge. Indeed, a new laser system can be coupled to the CyTOF device which allows for imaging mass cytometry (60). That is, the detection of metal-labeled antibodies, as in standard CyTOF analysis, but performed on tissue sections by using multiplexed ion beam imaging. This state of the art technology will allow, not only to assess a high range of markers at the same time, but also to obtain spatial resolution and warrant a very comprehensive analysis of the cell–cell interaction in the tumor microenvironment. New developments of the system should soon facilitate the analysis of tumor samples in a similar fashion, while gaining spatial resolution to better interrogate the role of spatial interactions in immunotherapy response (with high throughput) (61).

In conclusion, the use of NGS and CyTOF has great potential to discover novel biomarkers for immunotherapy and the studies discussed above show exciting promises, but need to be further validated before clinical application. New prospective trials with large cohorts could include these technologies as a biomarker discovery platform and could validate many of these findings. In parallel, new algorithms to integrate multiple high dimensional datasets are being developed for a combinatorial biomarker approach, which could use these existing datasets as a training model. As NGS is becoming a standard service in many clinics, the development of next generation biomarkers should ultimately improve the stratification of patients for immunotherapy and thereby extend OS for these patients.

## Author Contributions

SH, ML, and PC conceptualized the manuscript and oversaw all aspects of its completing including writing, figure design, and literature review.

## Conflict of Interest Statement

The authors declare that the research was conducted in the absence of any commercial or financial relationships that could be construed as a potential conflict of interest.
